# Comparative Physicochemical, Microbiological, Antioxidant, and Sensory properties of pre‐ and post‐fermented yoghurt enriched with olive leaf and its extract

**DOI:** 10.1002/fsn3.2704

**Published:** 2022-01-10

**Authors:** Samira Pourghorban, Linda Yadegarian, Maryam Jalili, Ladan Rashidi

**Affiliations:** ^1^ Faculty of Marine Science and Technology, Islamic Azad University, Northern Tehran Branch Tehran Iran; ^2^ Food Technology and Agricultural Products Research Center Standard Research Institute Iranian National Standards Organization (INSO) Karaj Iran

**Keywords:** enriched yoghurt, fermentation, olive leaf powder, sensory evaluation

## Abstract

This study investigated the comparative effect of yoghurt samples enriched with different concentrations of olive leaf powder (OLP) (0.1, 0.5, 1, and 1.5 mg/ml) and its extract (OLE) (0.5, 1, 3, and 5 mg/ml) on the microbiological, sensory, physicochemical, and antioxidant properties of pre‐ and post‐fermented samples during 21 days of storage. Sensory evaluation showed that concentrations lower than 1.5 and 5 mg/ml of OLP and OLE, respectively, were acceptable. Adding OLP or OLE did not have an influence on yoghurt starter culture bacteria (*p* > .05). All enriched samples significantly showed higher acidity and lower pH compared with control samples (*p* < .05). The most important polyphenols were oleuropein (from 0.132 to 0.224 and 0.373 to 0.413 mg/g for 0.5 and 3 mg/ml of OLE, from 0.194 to 0.321 mg/g and 0.413 to 1.280 mg/g for 0.5 and 1 mg/ml of OLP, respectively) and catechin (from 0.369 to 0.382 and 0.461 to 0.477 mg/g for 0.5 and 3 mg/ml of OLE, from 0.386 to 0.405 mg/g and 0.310 to 0.710 mg/g for 0.5 and 1 mg/ml of OLP, respectively) in enriched yoghurts. Adding OLP or OLE increased shelf life, antioxidant activity percentage (AA%), and total phenol content (TPC) of enriched samples (*p* < .05). During 21 days of storage of all samples, TPC, AA%, and pH decreased and TTA increased.

## Graphical abstract

In this study, olive leaf and olive leaf extract were used for the enrichment of yogurt. Olive leaf is a rich source of polyphenolic compounds and a healthy raw material. Organoleptic assessment of enriched yogurt samples with different concentrations of olive leaves and olive leaf extract was performed in the National Standard Organization of Iran. The organoleptic assessment of the new functional yogurt samples was performed by a testing panel, including 10 assessors, which evaluated the taste, color, and texture using a 1–5 scale, according to ISO 22935‐1, ‐2, and ‐3 methods. Physicochemical and microbiological tests were performed on novel yogurt formulations according to the method described by ISO 11869 and 7889, respectively. The viability of lactic acid bacteria and the physicochemical and organoleptic assessment of all yogurt samples were evaluated during 21 days of cold storage. Then, antioxidant activity (%AA), total phenol content (TPC), type and amount of polyphenol content of the enriched yogurt were examined during the 21‐day period. In addition, polyphenol compounds were detected and quantified by HPLC.

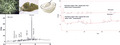

## 1 INTRODUCTION

Nowadays, incorporation of health‐promoting substances into diet and use of natural additives have attracted an increased interest. In addition, recycle of agricultural wastes and their use as dietary supplements are viewed as useful for economic and environmental reasons (Jaziri et al., 2009). Yoghurt is the most popular dairy product that is widely consumed as a functional food due to its good taste and high nutritional value (Reid et al., 2003). In recent years, some studies have been conducted on yoghurt supplementation with different herbs (Srivastava et al., 2015), certain vegetables (Najgebauer‐Lejko et al., 2014), fruit juice, peel and pulp (El‐Batawy et al., 2014; Gad et al., 2015; Selvamuthukumaran & Farhath, 2014), fish oil/γ‐oryzanol encapsulated by nanoemulsion (Rashidi, 2021; Zhong et al., 2018), milk proteins, including sodium caseinate, calcium caseinate, and milk protein concentrates (Delikanli & Ozcan, 2016), olive leaf (OL) encapsulated by nanoliposome (Tavakoli et al., 2018), and bioactive compounds, including oleuropein (Zoidou et al., 2014). Yoghurt supplemented with OL can be considered as a functional food. Olive leaves (OLs) are the farm by‐product of olive groves and are accumulated during pruning of olive trees (Molina‐Alcaide & Yanez‐Ruiz, 2008). Moreover, OLs can be obtained in high quantities in olive oil industries (10%). OL can be considered as a cheap and easily available natural source of different valuable compounds, including phenolic compounds, secoiridoids, flavonoids (Jemaiet et al., 2008), and high quantities of oleuropein (60–90 mg/g dry weight) (Ansari Dogaheh et al., 2011). Numerous studies (in vivo and in vitro) have demonstrated that oleuropein has a wide variety of biological activities, including antimicrobial, anti‐ischemic, antihypertensive, anti‐inflammatory, and anticarcinogenic properties (Zoidou et al., 2014).

In a related study, for the investigation of the effect of polyphenol‐enriched yoghurt on human health, 16 nonsmoking volunteers consumed either 400 g of olive polyphenol‐enriched yoghurt (containing 50 mg of encapsulated olive polyphenols) or 400 g of plain yoghurt every day for 2 weeks. The results indicated that consumption of the polyphenol‐enriched yoghurt may help reduce body weight, blood pressure, low‐density lipoprotein (LDL) cholesterol levels, and lipid peroxidation, and may promote growth of beneficial lactic acid bacteria (LAB) (Georgakouli et al., 2016). In this study, the aim was to enrich yoghurt, as it is a highly consumed product, with olive leaf and its extract (a rich source of polyphenols) in order to increase the functionality and shelf life of it. For this purpose, different concentrations of OLP and OLE were added to the yoghurt samples, pre‐ and post‐fermentation. Then, the sensory evaluation, survival of lactic acid bacteria, total titratable acidity (TTA) and pH changes, antioxidant activity percentage (AA%), total phenol content (TPC), and phenolic compounds of the yoghurt samples were determined.

## 2 MATERIAL AND METHODS

### 2.1 Materials

Fresh cows’ milk (3.6% fat) was obtained from a local milk farm in Karaj (Alborz, Iran). A starter culture (Yo‐MixTM 401 LYO) containing *Lactobacillus delbrueckii* subsp. *bulgaricus* (strain ATCC 1184) and *Streptococcus thermophilus* was purchased from Danisco (Niebüll, Germany). All chemical compounds used in the experiments, such as de Man, Rogosa and Sharpe (MRS) agar culture medium, MRS broth, and peptone, were purchased from Merck (Darmstadt, Germany). In addition, all of the chemicals, including acetonitrile (99.9%), orthophosphoric acid (85%), and methanol (99.99%), were of high‐performance liquid chromatography (HPLC) grade and purchased from Fisher Scientific.

### 2.2 Methods

#### 2.2.1 Preparation of OLs

Fresh young OLs were sampled from the *Roughani* variety (a native olive tree of Iran) in September 2017 from one of Fasa orchards at a latitude of 28.938300 (28°56′18″N) and a longitude of 53.648200 (53°38′54″E) located in Fars province of Iran. The OLs were washed several times with tap water and dried under vacuum at 25°C. The dried OLs were ground into a fine powder by a small electric grinder (Sanyo 260 W). Then, the olive leaf powder (OLP) was passed through a stainless steel sieve (the particle size of the OLP was approximately smaller than 50 microns) to obtain homogeneous and uniformly sized fine particles. The OLP was stored in a dark bottle at 25°C.

#### 2.2.2 Preparation of OLE

At first, 10 g of OLP and 50 ml of deionized water were mixed in a flask. The flask was put on a magnetic stirrer and stirred for 24 h in a dark place at 25°C. After stirring, the sample was filtered using a filter paper into a 50‐ml volumetric flask. The filtrate was passed through a sterile 0.22‐µm filter into a pre‐sterilized container. The filtrate was kept in a dark bottle at −20°C until further use.

#### 2.2.3 Preparation of yoghurt samples

The homogenized milk was pasteurized at 90°C for 5 min, cooled to 44 ± 1°C, and poured in the sterilized bottles (500 ml). For pre‐fermentation, defined concentrations of OLP (0.1, 0.5, 1, and 1.5 mg/ml) and OLE (0.5, 1, 3, and 5 mg/ml) were added to a series of sterilized bottles containing 500 ml of milk and thoroughly mixed. The bottles were labeled according to the concentration of added OLP or OLE. Then, 1 ml of starter culture solution (200 g of the starter was mixed with 1 liter of milk at room temperature) was added to each bottle. Then, they were incubated at 44 ± 1°C until the pH reached at 4.5 ± 0.1 (approximately 4–5 h).

For post‐fermentation, different concentrations of OLP or OLE were added to the series of sterilized bottles containing prepared yoghurt until the same concentrations of 0.1, 0.5, 1, and 1.5 mg/ml of OLP and 0.5, 1, 3, and 5 mg/ml of OLE were obtained in the final products. All samples were kept at 4°C, and the analysis was carried out after 1, 7, 14, and 21 days of cold storage. The control samples were prepared without OLP or OLE.

#### 2.2.4 Sensory analysis

Ten trained sensory panelists (comprised of five males and five females; 25–35 years old) evaluated randomly coded yoghurt samples. Texture, color, typical yoghurt flavor, and overall acceptability were evaluated on a 5‐point scale (1 = poor to 5 = excellent), while astringency, sourness, metallic taste, and bitterness were scored on a 5‐point scale (1 = low intensity to 5 = high intensity). The treatments with low sensory evaluation score (defined as not acceptable) were excluded from the study (2009, 2009, 2009 22935‐1, 2, and 3).

#### 2.2.5 Microbiological analysis

In order to investigate the effects of OLP and OLE on the viable bacteria of the enriched samples, the enumeration of characteristic microorganisms was performed by means of the colony‐count technique, according to the method described in ISO 7889 (2003). MRS agar medium with acidic pH was used for enumeration of *Lactobacillus delbrueckii subsp. bulgaricus*. Plates cultured under anaerobic conditions were incubated at 37°C for 72 h. However, M17 agar culture medium was used for enumeration of *Streptococcus thermophilus*. Plates cultured in aerobic conditions were incubated at 37°C for 48 h. Viable bacteria count in all entire samples was analyzed after 1, 7, 14, and 21 days of their cold storage at 4°C. The results are reported based on log colony‐forming units per gram (log cfu/g).

#### 2.2.6 pH and TTA

The pH of the yoghurt samples was measured with a pH meter (Jenwy 3510). All samples were homogenized in water at 1:9 ratios before pH determination (Amirdivani & Baba, 2011). The TTA of the new products was determined according to the ISO 11869: 2012 method. TTA was calculated as follows:

$$\text{Percentage}\;\text{of}\;\text{lactic}\;\text{acid}=\text{Dilution}\;\text{factor}\left(10\right)\times V_{\text{NaOH}}\times 0.1\times 0.009\times 100\%,$$

where *V* is the volume of NaOH required to neutralize the acid. All the analyses were performed in triplicate. The yoghurt samples were monitored for pH and TTA during cold storage for 1, 7, 14, and 21 days at 4°C.

#### 2.2.7 Extraction of phenolic compounds

The phenolic compounds were extracted from the enriched yoghurt samples according to the method described by Zoidou et al. (2014). Briefly, 1 g of yoghurt sample was diluted with 1 ml of distilled water, vortexed for 1 min, and sonicated in a water bath sonicator for 15 min. Then, the samples were centrifuged at 112 *g* for 5 min using a refrigerated centrifuge. The clear supernatant was separated and stored at −20°C for further use.

##### HPLC analysis

Determination and detection of polyphenol compounds in the yoghurt samples were carried out by HPLC, a system consisting of a quaternary pump coupled to a UV detector. A conventional reversed‐phase C18 column (250 mm × 4.6 mm, 5 μm) was used as the stationary phase. The gradient elution program was implemented using a system of two solvents as follows: solvent A consisted of water containing 0.2% H_3_PO_4_ (v/v), and solvent B was the mixture of methanol and acetonitrile (50:50 v/v). The flow rate was constant at 1 ml/min and chromatographic analysis was performed at 25°C. Phenolic compounds were detected at 280 nm. The HPLC gradient program was as follows: 0 min, 96% A; 40 min, 50% A; 45 min, 40% A; 60 min, 0% A, 70 min, 0% A; 72 min; 96% A. Identification of the eluting peaks of phenolic compounds was performed by comparing their retention time (tR) values and the corresponding UV spectra.

##### Standard solutions

The stock solution of 1 mg/ml was prepared from the phenolic compound standard. Then, by diluting appropriate volumes of the stock solution with methanol, working standards were obtained at lower concentrations (0.02, 0.04, 0.1, 0.2, and 0.4 mg/ml). Stock solution and working standards were stored in a refrigerator at 4°C.

#### 2.2.8 Determination of TPC

The TPC was determined by the colorimetric assay according to the method described by Mohammed and Manan (2008). A mixture containing yoghurt aliquot extract (200 μl), deionized water (800 μl of), and Folin–Ciocalteu reagent (100 μl) was prepared and incubated for 3 min at room temperature. Then, 300 μl of sodium carbonate (20%) was added to the mixture and incubated for 2 h at room temperature under dark conditions. The absorbance of the mixture was determined using UV–Vis spectrophotometer (Perkin Elmer Lmbda 25) at 765 nm. A blank sample was prepared with distilled water instead of the aliquot extract.

In addition, the gallic acid standard curve was prepared (from 0 to 100 mg/L) and TPC was expressed in mg of gallic acid equivalent/g dry matter. The analysis of samples was made in triplicate, and data are presented as mean ± standard deviation (SD).

#### 2.2.9 Determination of antioxidant activity (AA %)

The antioxidant activity percentage (AA %) of the yoghurt extract was determined according to the procedure reported by Robert et al. (1999). For this purpose, 2.5 mg of 2, 2‐diphenyl‐1‐picrylhydrazyl (DPPH) reagent was dissolved in 100‐ml methanol. This stock solution was kept in a dark place at ambient temperature. A quantity of 0.1 ml of yoghurt extract was mixed with 3.9 ml of methanolic DPPH solution. The control sample was prepared with the same volume of methanol and deionized water instead of the sample. Absorbance was measured at 515 nm by a UV–Vis spectrophotometer (Perkin Elmer Lmbda 25). The measurements were carried out in triplicate.

##### Data analysis

Statistical analysis was performed with SPSS Version 11.0 statistic software package and Duncan's test for mean comparison was adopted to highlight significant differences among the yoghurt samples. Data were significant if the *p*‐value was found to be <0.05. Data are expressed as means ± standard deviation (SD).

## 3 RESULTS

### 3.1 Sensory analysis

Sensory properties of the final products play the most important role in their daily consumptions. Therefore, organoleptic assessment was performed by 10 trained panelists at the Institute of Standards and Industrial Research of Iran (ISIRI) according to the ISO 22935‐1 method. Results of the organoleptic assessment of the enriched yoghurt samples are presented in Table 1.

**TABLE 1:** Results of organoleptic assessment of the enriched yoghurt samples with OLP or OLE, pre‐ and post‐fermentation, during storage at 4°C (values are means ± SD)

Enriched yoghurt samples with OLP or OLE	Concentration of OLP/OLE (mg/ml)	Code of assessors										Mean ± SD
		1	2	3	4	5	6	7	8	9	10	
		Results of magnitude of the deviation in enriched yoghurt products from the pre‐established sensory specifications										
OLE added pre‐ fermentation	0.5	5 ± 0.01^Aa^	5 ± 0.03^Aa^	5 ± 0.00^Aa^	5 ± 0.01^Aa^	5 ± 0.02^Aa^	5 ± 0.01^Aa^	5 ± 0.00^Aa^	5 ± 0.01^Aa^	5 ± 0.02^Aa^	5 ± 0.02^Aa^	5.0 ± 0.13^Aa^
	1	5 ± 0.02^Aa^	5 ± 0.03^Aa^	5 ± 0.01^Aa^	5 ± 0.02^Aa^	5 ± 0.01^Aa^	5 ± 0.01^Aa^	5 ± 0.05^Aa^	5 ± 0.02^Aa^	5 ± 0.01^Aa^	5 ± 0.03^Aa^	5.0 ± 0.21^Aa^
	3	5 ± 0.01^Aa^	4 ± 0.02^Bb^	4 ± 0.05^Bb^	4 ± 0.03^Bb^	4 ± 0.03 ^Bb^	5 ± 0.01^Aa^	4 ± 0.12 ^Bb^	4 ± 0.01^Bb^	3 ± 0.12 ^Cc^	4 ± 0.01 ^Bb^	4.1 ± 0.41^Bb^
	5	2 ± 0.0^Bd^	2 ± 0.05^Dd^	2 ± 0.0^Dd^	3 ± 0.03^Cc^	3 ± 0.05^Cc^	3 ± 0.01^Cc^	2 ± 0.03^Dd^	2 ± 0.02^Dd^	2 ± 0.01^Dd^	3 ± 0.03^Cc^	2.4 ± 0.28^Ddc^
OLE added post‐ fermentation	0.5	5 ± 0.01^Aa^	5 ± 0.02^Aa^	5 ± 0.03^Aa^	5 ± 0.01^Aa^	5 ± 0.01^Aa^	5 ± 0.01^Aa^	5 ± 0.02^Aa^	5 ± 0.01^Aa^	5 ± 0.06^Aa^	5 ± 0.04^Aa^	5.0 ± 0.22^Aa^
	1	4 ± 0.02^Bb^	4 ± 0.02^Bb^	4 ± 0.02^Bb^	5 ± 0.01^Aa^	5 ± 0.03^Aa^	5 ± 0.03^a^	5 ± 0.02^Aa^	4 ± 0.03^Bb^	4 ± 0.01^Bb^	4 ± 0.06^Bb^	4.6 ± 0.25^ABab^
	3	4 ± 0.02^Bb^	3 ± 0.02^Cc^	4 ± 0.05^Bb^	4 ± 0.05^Bb^	5 ± 0.03^Aa^	4 ± 0.03 ^Bb^	4 ± 0.02^Bb^	4 ± 0.05^Bb^	4 ± 0.03^Bb^	4 ± 0.09 ^Bb^	4.4 ± 0.39^ABba^
	5	3 ± 0.03^Cc^	3 ± 0.02^Cc^	3 ± 0.10 ^Cc^	2 ± 0.07^Dd^	2 ± 0.04^Dd^	3 ± 0.10^Cc^	3 ± 0.03^Cc^	3 ± 0.05^Cc^	2 ± 0.10^Dd^	2 ± 0.02^Dd^	2.5 ± 0.56^DCdc^
OLP added pre‐ fermentation	0.1	5 ± 0.01^Aa^	5 ± 0.01^Aa^	4 ± 0.04^Bb^	4 ± 0.03^Bb^	4 ± 0.07^Bb^	5 ± 0.02^Aa^	5 ± 0.04^Aa^	5 ± 0.02^Aa^	4 ± 0.08^Bb^	5 ± 0.07^Aa^	4.6 ± 0.37^ABab^
	0.5	4 ± 0.02^Bb^	5 ± 0.03^Aa^	4 ± 0.08^Bb^	4 ± 0.02^Bb^	5 ± 0.03^Aa^	4 ± 0.03 ^Bb^	4 ± 0.03^Bb^	5 ± 0.05^Aa^	4 ± 0.05^Bb^	5 ± 0.08^Aa^	4.4 ± 0.42^ABab^
	1	3 ± 0.01^Cc^	4 ± 0.04^Bb^	3 ± 0.05 ^Cc^	4 ± 0.05^Bb^	3 ± 0.03^Cc^	3 ± 0.06 ^Cc^	4 ± 0.04^Bb^	4 ± 0.08^Bb^	3 ± 0.09^Cc^	3 ± 0.10^Cc^	3.4 ± 0.55^BCbc^
	1.5	1 ± 0.05^Ee^	1 ± 0.02^Ee^	1 ± 0.02^Ee^	1 ± 0.04^Ee^	1 ± 0.02^Ee^	1 ± 0.02^Ee^	1 ± 0.02^Ee^	1 ± 0.06^Ee^	1 ± 0.30^Ee^	1 ± 0.14^Ee^	1.0 ± 0.69^Ee^
OLP added Post‐ fermentation	0.1	5 ± 0.03^Aa^	4 ± 0.03^Bb^	5 ± 0.05^Aa^	4 ± 0.03^Bb^	4 ± 0.01^Bb^	5 ± 0.08^a^	4 ± 0.04^Bb^	4 ± 0.05^Bb^	5 ± 0.01^Aa^	4 ± 0.02^Bb^	4.4 ± 0.35^ABba^
	0.5	4 ± 0.01^Bb^	4 ± 0.01^Bb^	3 ± 0.06^Cc^	3 ± 0.04^Cc^	3 ± 0.04^Cc^	4 ± 0.07^Bb^	3 ± 0.10^Cc^	4 ± 0.05^Bb^	4 ± 0.06^Bb^	4 ± 0.02^Bb^	3.6 ± 0.64^BCbc^
	1	3 ± 0.01^Cc^	3 ± 0.05^Cc^	3 ± 0.04 ^Cc^	2 ± 0.01^Dd^	2 ± 0.02^Dd^	3 ± 0.03^Cc^	2 ± 0.05^Dd^	2 ± 0.05^Dd^	3 ± 0.06^Cc^	2 ± 0.04^Dd^	2.5 ± 0.36^CDcd^
	1.5	2 ± 0.02^Dd^	1 ± 0.04^Ee^	1 ± 0.05^Ee^	1 ± 0.05^Ee^	1 ± 0.05^Ee^	2 ± 0.05^Dd^	1 ± 0.05^Ee^	1 ± 0.07^Ee^	2 ± 0.10^Dd^	2 ± 0.07^Dd^	1.42 ± 0.54^DEde^

Note

Data are expressed as the means ± SD for three replicates. ^abcd^Different superscripts in the same row (*p* < .05). ^ABCD^Different superscripts in the same column (*p* < .05).

It was observed that concentrations of 5 mg/ml of OLE and 1.5 mg/ml of OLP were not accepted, pre‐ or post‐fermentation, due to unacceptable sensory specifications, including color, bitter flavor, and undesirable texture (*p* < .05). The results showed that increase of OLE concentration to 5 mg/ml was made to sense a potential flavor of olive leaf.

### 3.2 Microbiological analysis

Viable bacteria counts were performed on days 1, 7, 14, and 21 in triplicate. Table 2 shows the enumeration of *L*. *delbrueckii* subsp. *bulgaricus* the yoghurt samples. The results showed that there was no significant effect on the total count of *L*. *delbrueckii* subsp. *bulgaricus* compared with control after 1, 7, 14, or 21 days of storage (*p* > .05). The viable counts of *L*. *delbrueckii* subsp. *bulgaricus* increased slightly with increase in OLE or OLP concentration, pre‐ or post‐fermentation. In all samples, the viable counts of *L*. *delbrueckii* subsp. *bulgaricus* decreased slightly or remained constant during the 21‐day storage at 4°C (*p* > .05).

**TABLE 2:** Viability of *Lactobacillus bulgaricus* subsp. *delbrueckii* (Log cfu/ml), pre‐ and post‐fermentation, during storage at 4°C (values are means ± SD)

Concentrations of added OLE							
Days	0	0.5 mg/ml of OLE (pre‐fermentation)	1.0 mg/ml of OLE (pre‐fermentation)	3.0 mg/ml of OLE (pre‐fermentation)	0.5 mg/ml of OLE (post‐fermentation)	1.0 mg/ml of OLE (post‐fermentation)	3.0 mg/ml of OLE (post‐fermentation)
1	6.755 ± 0.05^Aa^	6.763 ± 0.03^Aa^	6.778 ± 0.03^Ab^	6.799 ± 0.02^Ac^	6.763 ± 0.02^Aa^	6.792 ± 0.01^Ac^	6.826 ± 0.02^Ac^
7	6.633 ± 0.02^Ba^	6.653 ± 0.04^Ba^	6.672 ± 0.02^Ba^	6.699 ± 0.04^Ba^	6.653 ± 0.01^Ba^	6.681 ± 0.04^Bb^	6.724 ± 0.06^Bc^
14	6.322 ± 0.04^Ca^	6.361 ± 0.05^Cb^	6.380 ± 0.05^Cc^	6.398 ± 0.01^Cb^	6.362 ± 0.04^Cb^	6.398 ± 0.06^Cc^	6.462 ± 0.07^Cb^
21	5.924 ± 0.06^Db^	6.361 ± 0.02^Ca^	5.944 ± 0.03^Db^	5.954 ± 0.06^Db^	6.362 ± 0.05^Ca^	5.914 ± 0.03^Db^	5.959 ± 0.05^Db^

Concentrations of added OLP							
Days	0	0.1 mg/ml of OLP (pre‐fermentation)	0.5 mg/ml of OLP (pre‐fermentation)	1.0 mg/ml of OLP (pre‐fermentation)	0.1 mg/ml of OLP (post‐fermentation)	0.5 mg/ml of OLP (post‐fermentation)	1.0 mg/ml of OLP (post‐fermentation)
1	6.755 ± 0.05^Aa^	6.740 ± 0.05^Aa^	6.771 ± 0.02^Aa^	6.806 ± 0.05^Ab^	6.763 ± 0.07^Aa^	6.799 ± 0.01^Ac^	6.839 ± 0.02^Ab^
7	6.633 ± 0.02^Ba^	6.643 ± 0.04^Ba^	6.672 ± 0.05^Bb^	6.672 ± 0.06^Bb^	6.462 ± 0.05^Bc^	6.681 ± 0.09^Bb^	6.672 ± 0.06^Bb^
14	6.322 ± 0.04^Ca^	6.322 ± 0.03^Ca^	6.414 ± 0.07^Cb^	6.431 ± 0.02^Cb^	6.361 ± 0.03^Ca^	6.414 ± 0.04^Cb^	6.301 ± 0.04^Ca^
21	5.924 ± 0.06^Da^	5.923 ± 0.07^Da^	5.949 ± 0.05^Da^	5.892 ± 0.07^Db^	5.929 ± 0.05^Da^	5.954 ± 0.05^Da^	5.929 ± 0.07^Da^

Note

Data are expressed as the means ± SD for three replicates. ^abcd^Different superscripts in the same row (*p* < .05). ^ABCD^Different superscripts in the same column (*p* < .05).

The enumeration of *S*. *thermophilus* in the enriched yoghurt products, as well as in control samples, during storage for 21 days is presented in Table 3. It was observed that adding OLP or OLE did not significantly affect the total count of the *S*. *thermophilus* in the enriched yoghurt samples in comparison with control yoghurt samples at the end of each studied period (*p* > .05). Also, it was observed that the colonies of the *S*. *thermophilus* had existed in all enriched samples over 21 days of storage and no significant decrease was observed after the 21‐day storage (*p* > .05). The findings of the current study revealed that *S*. *thermophilus* bacteria were more stable in comparison with L. *delbrueckii* subsp. *bulgaricus* after 21 days of storage in all enriched yoghurt samples. In addition, the viable counts of *S*. *thermophilus* increased slightly or remained constant with increase of OLP or OLE concentration, pre‐ or post‐fermentation.

**TABLE 3:** Viability of *Streptococcus thermophilus* (Log cfu/ml), pre‐ and post‐fermentation, during storage at 4°C (values are means ± SD)

Concentrations of added OLE							
Days	0	0.5 mg/ml of OLE (pre‐fermentation)	1.0 mg/ml of OLE (pre‐fermentation)	3.0 mg/ml of OLE (pre‐fermentation)	0.5 mg/ml of OLE (post‐fermentation)	1.0 mg/ml of OLE (post‐fermentation)	3.0 mg/ml of OLE (post‐fermentation)
1	6.857 ± 0.07^Aa^	6.899 ± 0.01^Ab^	6.945 ± 0.07^Ab^	6.942 ± 0.05^Ab^	6.863 ± 0.05^Aa^	6.892 ± 0.03^Aa^	6.908 ± 0.04^Aa^
7	6.844 ± 0.02^Ba^	6.887 ± 0.09^Aab^	6.891 ± 0.04^Ab^	6.895 ± 0.04^Ba^	6.954 ± 0.01^Ba^	6.982 ± 0.02^Ba^	6.996 ± 0.05^Bc^
14	6.869 ± 0.03^Aa^	6.886 ± 0.011^Ab^	6.888 ± 0.05^Bb^	6.922 ± 0.03^Cb^	6.886 ± 0.05^Ab^	6.919 ± 0.07^Cb^	6.919 ± 0.04^Ab^
21	6.633 ± 0.05^Ca^	6.613 ± 0.07^Bb^	6.603 ± 0.03^Cb^	6.616 ± 0.09^Cb^	6.633 ± 0.06^Ca^	6.662 ± 0.03^Dc^	6.707 ± 0.07^Cd^

Concentrations of added OLP							
Days	0	0.1 mg/ml of OLP (pre‐fermentation)	0.5 mg/ml of OLP (pre‐fermentation)	1.0 mg/ml of OLP (pre‐fermentation)	0.1 mg/ml of OLP (post‐fermentation)	0.5 mg/ml of OLP (post‐fermentation)	1.0 mg/ml of OLP (post‐fermentation)
1	6.857 ± 0.07^Aa^	6.845 ± 0.02^Aa^	6.875 ± 0.03^Aa^	6.892 ± 0.02^Ab^	6.857 ± 0.05^Aa^	6.880 ± 0.01^Ab^	6.924 ± 0.03^Ac^
7	6.944 ± 0.02^Ba^	6.944 ± 0.05^Ba^	6.919 ± 0.01^Bc^	6.959 ± 0.04^B^	6.949 ± 0.03^Ba^	6.973 ± 0.06^Bd^	6.986 ± 0.05^Be^
14	6.869 ± 0.03^Ba^	6.869 ± 0.07^Aa^	6.875 ± 0.05^Aa^	6.903 ± 0.05^Bb^	6.880 ± 0.01^Aa^	6.919 ± 0.01^Cc^	6.919 ± 0.01^Ac^
21	6.633 ± 0.05^Ca^	6.643 ± 0.03^Ca^	6.568 ± 0.07^Cb^	6.756 ± 0.08^Cc^	6.643 ± 0.05^Ca^	6.653 ± 0.04^Da^	6.763 ± 0.07^Cc^

Note

Data are expressed as the means ± SD for three replicates. ^abcd^Different superscripts in the same row (*p* < .05). ^ABCD^Different superscripts in the same column (*p* < .05). ^NS^not significant.

### 3.3 pH and TTA values

pH and TTA are known key factors that impact the shelf life and acceptability of dairy products. Table 4 shows the pH values of yoghurt samples enriched with OLP or OLE, which were determined every week over a period of 21 days. The results showed that pH values of all products significantly dropped during the storage period (*p* < .05). Also, the addition of OLP at different concentrations, pre‐ or post‐ fermentation, had a slight effect on the pH of enriched samples when compared to that of plain yoghurt samples (*p* > .05). Increase of OLP or OLE concentration resulted in a slight decrease in the pH of yoghurt samples, pre‐ or post‐fermentation. The pH values of all products dropped (*p* < .05) due to the activity of the lactic acid bacteria during the storage. Table 5 shows the TTA values of plain and enriched yoghurt samples. The results show an increase in TTA values of all enriched and control samples during the storage at 4^◦^C. The addition of OLP or OLE, pre‐ and post‐fermentation, increased the TTA values of all enriched samples when compared to those of plain yoghurt samples (*p* < .05). Moreover, the TTA value of enriched yoghurt samples increased as OLP or OLE concentrations in the yoghurt samples increased. In post‐fermentation, the highest and lowest of TTA values, respectively, belonged to the enriched yoghurt sample with 1 mg/ml of OLP and plain yoghurt during the cold storage. It was observed that the addition of OLP or OLE pre‐fermentation had a slight effect on the TTA value in the enriched samples, when compared to post‐fermentation.

**TABLE 4:** pH values of yoghurt samples, pre‐ and post‐fermentation, during storage at 4°C **(**values are means ± SD)

Concentrations of added OLE							
Days	0	0.5 mg/ml of OLE (pre‐fermentation)	1.0 mg/ml of OLE (pre‐fermentation)	3.0 mg/ml of OLE (pre‐fermentation)	0.5 mg/ml of OLE (post‐fermentation)	1.0 mg/ml of OLE (post‐fermentation)	3.0 mg/ml of OLE (post‐fermentation)
1	4.570 ± 0.05^Aa^	4.540 ± 0.02^Ab^	4.530 ± 0.09^Ab^	4.530 ± 0.07^Ab^	4.570 ± 0.02^Aa^	4.550 ± 0.08^Ab^	4.550 ± 0.09^Ab^
7	4.450 ± 0.02^Ba^	4.440 ± 0.05^Ba^	4.420 ± 0.04^Ba^	4.410 ± 0.06^Ba^	4.430 ± 0.08^Ba^	4.400 ± 0.06^Ba^	4.410 ± 0.04^Ba^
14	4.350 ± 0.04^Ca^	4.350 ± 0.09^Ca^	4.330 ± 0.02^Ca^	4.320 ± 0.02^Ca^	4.340 ± 0.05^Ca^	4.300 ± 0.01^Ca^	4.320 ± 0.05^Ca^
21	4.300 ± 0.06^Ca^	4.300 ± 0.04^Ca^	4.290 ± 0.05^Ca^	4.270 ± 0.05^Ca^	4.300 ± 0.01^Ca^	4.270 ± 0.03^Ca^	4.260 ± 0.04^Ca^

Concentrations of added OLP							
Days	0	0.1 mg/ml of OLP (pre‐fermentation)	0.5 mg/ml of OLP (pre‐fermentation)	1.0 mg/ml of OLP (pre‐fermentation)	0.1 mg/ml of OLP (post‐fermentation)	0.5 mg/ml of OLP (post‐fermentation)	1.0 mg/ml of OLP (post‐fermentation)
1	4.570 ± 0.05^Aa^	4.550 ± 0.05^Aa^	4.520 ± 0.02^Ab^	4.510 ± 0.05^Aa^	4.570 ± 0.02^Aa^	4.550 ± 0.02^Aa^	4.550 ± 0.07^Aa^
7	4.450 ± 0.02^Ba^	4.440 ± 0.04^Bb^	4.440 ± 0.05^Ab^	4.400 ± 0.06^Bb^	4.430 ± 0.05^Bb^	4.440 ± 0.05^Bb^	4.400 ± 0.03^Bb^
14	4.350 ± 0.0^4Ba^	4.340 ± 0.03^Ba^	4.330 ± 0.07^Ba^	4.310 ± 0.02^Ba^	4.330 ± 0.07^Ba^	4.310 ± 0.07^Ba^	4.300 ± 0.04^Ba^
21	4.300 ± 0.06^Ba^	4.310 ± 0.07^Ba^	4.270 ± 0.05^Ba^	4.260 ± 0.07^Ba^	4.300 ± 0.05^Ba^	4.280 ± 0.05^Ba^	4.270 ± 0.07^Ba^

Note

Data are expressed as the means ± SD for three replicates. ^abcd^Different superscripts in the same row (*p* < .05). ^ABCD^Different superscripts in the same column (*p* < .05).

**TABLE 5:** TTA values of yoghurt samples, pre‐ and post‐fermentation, during storage at 4°C (values are means ± SD)

Concentrations of added OLE							
Days	0	0.5 mg/ml of OLE (pre‐fermentation)	1.0 mg/ml of OLE (pre‐fermentation)	3.0 mg/ml of OLE (pre‐fermentation)	0.5 mg/ml of OLE (post‐fermentation)	1.0 mg/ml of OLE (post‐fermentation)	3.0 mg/ml of OLE (post‐fermentation)
1	0.932 ± 0.06^Aa^	0.932 ± 0.04^Aa^	0.937 ± 0.05^Aa^	0.937 ± 0.03^Aa^	0.920 ± 0.04^Aa^	0.924 ± 0.01^Aa^	0.927 ± 0.05^Aa^
7	0.975 ± 0.05^Ba^	0.974 ± 0.07^Ba^	0.980 ± 0.06^Ba^	0.983 ± 0.06^Ba^	0.975 ± 0.05^Ba^	0.980 ± 0.05^Ba^	0.985 ± 0.03^Ba^
14	1.010 ± 0.04^Ba^	1.010 ± 0.02^Ba^	1.015 ± 0.09^Ca^	1.020 ± 0.05^Ba^	1.010 ± 0.08^Ba^	1.025 ± 0.08^Ba^	1.022 ± 0.06^Ba^
21	0.934 ± 0.03^Aa^	0.927 ± 0.02^Aa^	1.034 ± 0.05^Cb^	1.040 ± 0.03^Bb^	0.920 ± 0.04^Ba^	1.038 ± 0.06^Bb^	1.045 ± 0.05^Bb^

Concentrations of added OLP							
Days	0	0.1 mg/ml of OLP (pre‐fermentation)	0.5 mg/ml of OLP (pre‐fermentation)	1.0 mg/ml of OLP (pre‐fermentation)	0.1 mg/ml of OLP (post‐fermentation)	0.5 mg/ml of OLP (post‐fermentation)	1.0 mg/ml of OLP (post‐fermentation)
1	0.932 ± 0.06^Aa^	0.927 ± 0.02^Aa^	0.940 ± 0.05^Aa^	0.945 ± 0.03^Aa^	0.920 ± 0.04^Aa^	0.927 ± 0.02^Aa^	0.927 ± 0.05^Aa^
7	0.975 ± 0.05^Ba^	0.973 ± 0.05^Ba^	0.981 ± 0.08^Ba^	0.987 ± 0.06^Ba^	0.975 ± 0.05^Ba^	0.988 ± 0.04^Ba^	0.990 ± 0.03^Ba^
14	1.015 ± 0.04^Ca^	1.010 ± 0.02^Ca^	1.018 ± 0.07^Ca^	1.025 ± 0.05^Ca^	1.010 ± 0.08^Ca^	1.023 ± 0.07^Ca^	1.027 ± 0.06^Ca^
21	1.034 ± 0.03^Ca^	1.020 ± 0.03^Ca^	1.040 ± 0.05^Ca^	1.044 ± 0.09^Ca^	1.030 ± 0.02^Ca^	1.037 ± 0.08^Ca^	1.040 ± 0.05^Ca^

Note

Data are expressed as the means ± SD for three replicates. ^abcd^Different superscripts in the same row (*p* < .05). ^ABCD^Different superscripts in the same column (*p* < .05).

### 3.4 Identification of phenolic compounds

The limits of detection (LOD) and quantitation (LOQ) were obtained for vanillin, vanillic acid, caffeic acid, oleuropein, apigenin, luteolin, tyrosol, and catechin. It was observed that the LOD and LOQ ranged approximately from 10 to 30 and 30 to 90 µg/g, respectively. Table 6 shows the quantities of phenolic compounds in the enriched yoghurt samples with OLP and OLE, pre‐ and post‐ fermentation, after 1 day of storage at 4°C. Results showed that oleuropein, catechin, and tyrosol were the most abundant phenolic compounds detected by HPLC in the enriched yoghurt samples. The maximum amounts of oleuropein (1.280 mg/ml) and catechin (0.710 mg/ml) were found in the yoghurt sample enriched with 1 mg/ml of OLP post‐fermentation. The lower amounts of oleuropein and other phenolic compounds were observed when OLE and OLP were added pre‐fermentation. Results showed that the yoghurt sample enriched with OLP (both pre‐ and post‐fermentation) contained higher amounts of phenolic compounds compared to yoghurt samples enriched with OLE. Moreover, by increasing the OLP and OLE concentrations, the amounts of entire phenolic compound increased.

**TABLE 6:** Phenolic compounds in the enriched yoghurt samples (mg/g ± SD)

Phenolic Compounds	0.5 mg/ml (OLE pre‐fermentation)	0.5 mg/ml (OLE post‐fermentation)	0.5 mg/ml (OLP pre‐fermentation)	0.5 mg/ml (OLP post‐fermentation)
Gallic acid	0.012 ± 0.001^a^	0.026 ± 0.002^b^	0.030 ± 0.001^c^	0.05 ± 0.002^d^
Tyrosol	0.075 ± 0.024^a^	0.101 ± 0.005^b^	0.088 ± 0.008^a^	0.251 ± 0.063^c^
Catechin	0.369 ± 0.059^a^	0.382 ± 0.016^b^	0.386 ± 0.022^b^	0.405 ± 0.005 ^c^
Caffeic acid	0.091 ± 0.029^a^	0.103 ± 0.007^a^	0.077 ± 0.000^b^	0.121 ± 0.013^c^
Vanillin	0.094 ± 0.007^a^	0.113 ± 0.009^b^	0.089 ± 0.012^c^	0.094 ± 0.004^a^
Oleuropein	0.132 ± 0.036^a^	0.224 ± 0.008^b^	0.194 ± 0.023^c^	0.321 ± 0.003^d^
Apigenin	0.061 ± 0.003^a^	0.071 ± 0.007^b^	0.112 ± 0.003^c^	0.227 ± 0.002^d^
Ferulic acid	0.011 ± 0.001^a^	0.132 ± 0.003^b^	0.027 ± 0.004^c^	0.021 ± 0.005^d^

Phenolic compounds	3 mg/ml (OLE pre‐fermentation)	3 mg/ml (OLE post‐fermentation)	1 mg/ml (OLP pre‐fermentation)	1 mg/ml (OLP post‐fermentation)
Gallic Acid	0.046 ± 0.019^a^	0.062 ± 0.014^ab^	0.083 ± 0.005^b^	0.074 ± 0.005^c^
Tyrosol	0.122 ± 0.008^bc^	0.131 ± 0.006^a^	0.100 ± 0.003^b^	0.143 ± 0.001^c^
Catechin	0.461 ± 0.002^a^	0.477 ± 0.064^b^	0.310 ± 0.006^c^	0.710 ± 0.054^d^
Caffeic Acid	0.010 ± 0.000^a^	0.271 ± 0.031^b^	0.140 ± 0.001^c^	0.151 ± 0.033^d^
Vanillin	0.092 ± 0.034^a^	0.124 ± 0.020^b^	0.152 ± 0.012^c^	0.157 ± 0.003^c^
Oleuropein	0.373 ± 0.017^a^	0.413 ± 0.009^b^	0.413 ± 0.003^b^	1.280 ± 0.099^c^
Apigenin	0.010 ± 0.001^a^	0.062 ± 0.007^b^	0.100 ± 0.005^c^	0.110 ± 0.001^d^
Ferulic Acid	0.132 ± 0.006^a^	0.011 ± 0.001^b^	0.071 ± 0.003^ab^	0.080 ± 0.004^c^

Note

Different letters in the same row show significant differences (*p* < .05).

### 3.5 Evaluation of TPC in the enriched yoghurt

Results of TPC determined in the enriched and plain yoghurt samples during 0, 7, 14, and 21 days are shown in Table 7. The results showed that the TPC of all enriched yoghurt samples decreased during 21 days of cold storage (*p* < .05). On the first day, the maximum TPC values of enriched samples with 1 mg/ml OLP and 3 mg/ml of OLE obtained were 65.55 mg GAE/ml and 53.97 mg GAE/ml in the post‐fermentation, respectively. In all samples, the levels of TPC were higher when OLP or OLE was added post‐fermentation in comparison with those of pre‐fermentation. A positive relationship between the concentration of OLP or OLE and TPC was observed. Besides the starter culture activities, the time, and temperature of fermentation might have affected this condition.

**TABLE 7:** TPC values of yoghurt samples (mg GAE/ml), pre‐ and post‐fermentation, during storage at 4°C (values are means ± SD)

Concentrations of added OLE							
Days	0	0.5 mg/ml of OLE (pre‐fermentation)	1.0 mg/ml of OLE (pre‐fermentation)	3.0 mg/mL of OLE (pre‐fermentation)	0.5 mg/mL of OLE (post‐fermentation)	1.0 mg/mL of OLE (post‐fermentation)	3.0 mg/mL of OLE (post‐fermentation)
1	40.26 ± 2.54^Aa^	45.12 ± 1.79^Aa^	46.00 ± 1.15^Aa^	49.00 ± 0.98^Ab^	46.00 ± 0.98^Aa^	48.00 ± 1.97^Ab^	53.97 ± 2.62^Ab^
7	36.57 ± 1.24^Ba^	39.00 ± 0.72^Ba^	43.00 ± 1.35^Aa^	45.57 ± 1.61^Aa^	43.00 ± 1.01^Aa^	46.28 ± 1.41^Aab^	49.85 ± 1.48^Ab^
14	25.05 ± 2.24^Ca^	29.50 ± 0.70^Cb^	33.01 ± 2.51^Bc^	36.00 ± 1.47^Bc^	29.90 ± 2.16^Bb^	34.02 ± 2.52^Bc^	38.02 ± 1.47^Bd^
21	20.12 ± 1.06^Ca^	25.50 ± 1.67^Cb^	32.03 ± 1.39^Bc^	33.50 ± 1.55^Bc^	26.70 ± 1.05^Bb^	32.38 ± 0.56^Bc^	33.85 ± 1.61^Cc^

Concentrations of added OLP							
Days	0	0.1 mg/ml of OLP (pre‐fermentation)	0.5 mg/ml of OLP (pre‐fermentation)	1.0 mg/ml of OLP (pre‐fermentation)	0.1 mg/ml of OLP (post‐fermentation)	0.5 mg/ml of OLP (post‐fermentation)	1.0 mg/ml of OLP (post‐fermentation)
1	40.26 ± 2.54^Aa^	42.50 ± 1.15^Aa^	53.43 ± 3.44^Ab^	58.78 ± 2.03^Ac^	44.90 ± 1.76^Aa^	58.46 ± 1.72^Ac^	65.55 ± 1.73^Ad^
7	36.57 ± 1.24^Ba^	41.00 ± 1.06^Ab^	47.93 ± 2.18^Ac^	51.30 ± 2.01^Ac^	41.30 ± 1.68^Ab^	50.89 ± 2.17^Ac^	59.55 ± 0.41^Bd^
14	25.05 ± 2.24^Ca^	30.00 ± 0.75^Bb^	33.43 ± 2.82^Bb^	36.00 ± 2.43^Bc^	31.50 ± 1.70^Bb^c	35.50 ± 0.78^Bc^	39.50 ± 1.37^Bd^
21	20.12 ± 1.06^Ca^	26.72 ± 2.09^Cb^	30.55 ± 2.24 ^Bc^	33.04 ± 2.80^Bc^	27.50 ± 1.56^Bb^	32.30 ± 1.74^Bc^	33.70 ± 1.85^Cbc^

Note

Data are expressed as the means ± SD for three replicates. ^abcd^Different superscripts in the same row (*p* < .05). ^ABCD^Different superscripts in the same column (*p* < .05).

### 3.6 Evaluation of antioxidant activity (AA%)

DPPH assay was performed to determine the antioxidant activity percentage (AA%) of the yoghurt samples. The results showed that the antioxidant activity values of enriched samples were higher than those of control samples (Table 8). The antioxidant activity values of all enriched products declined during the cold storage period, but remained in the highest amounts during the first week (*p* < .05). Also, in post‐fermentation, the values of antioxidant activity of enriched yoghurt samples with OLP or OLE were higher than those of pre‐fermentation. Maximum antioxidant activity values were observed in yoghurt samples enriched with 1 mg/ml OLP and 3 mg/ml OLE after fermentation (58.55% and 46.97%, respectively). Also, the AA% in the post‐fermentation enriched yoghurt samples with OLP or OLE was higher than those of the pre‐fermentation prepared samples. It seems that this condition is due to the instability of phenolic compounds.

**TABLE 8:** Antioxidant activity percentages (AA% of yoghurt samples) pre‐ and post‐fermentation, during storage at 4°C (values are means ± SD)

Concentrations of added OLE							
Days	0	0.5 mg/ml of OLE (pre‐fermentation)	1.0 mg/ml of OLE (pre‐fermentation)	3.0 mg/ml of OLE (pre‐fermentation)	0.5 mg/ml of OLE (post‐fermentation)	1.0 mg/ml of OLE (post‐fermentation)	3.0 mg/ml of OLE (post‐fermentation)
1	25.70 ± 1.35^Aa^	41.12 ± 1.79^Ab^	42.00 ± 2.00^Ac^	42.90 ± 1.13^Abc^	46.00 ± 0.98^Adc^	46.50 ± 2.97^Abc^	46.97 ± 2.62^Adc^
7	21.30 ± 1.2^Ba^	39.00 ± 0.72^Bc^	39.50 ± 2.35^Bb^	40.10 ± 1.81^Ac^	43.00 ± 1.01^Ac^	43.11 ± 1.15^Ac^	43.85 ± 2.48^Ac^
14	19.05 ± 0.62^Ba^	29.50 ± 0.70^Cb^	28.05 ± 2.05^Cb^	36.25 ± 1.77^Bc^	29.90 ± 2.16^Bb^	30.02 ± 1.90^Bb^	38.50 ± 1.47^Bc^
21	14.12 ± 1.47^Ca^	25.50 ± 1.67^Db^	26.23 ± 1.45^Cb^	35.00 ± 1.45^Bc^	26.70 ± 1.05^Bb^	29.00 ± 1.56^Bb^	37.50 ± 0.61^Bc^

Concentrations of added OLP							
Days	0	0.1 mg/ml of OLP (pre‐fermentation)	0.5 mg/ml of OLP (pre‐fermentation)	1.0 mg/ml of OLP (pre‐fermentation)	0.1 mg/ml of OLP (post‐fermentation)	0.5 mg/ml of OLP (post‐fermentation)	1.0 mg/ml of OLP (post‐fermentation)
1	25.70 ± 1.35^Aa^	42.50 ± 1.15^Ab^	43.43 ± 3.44^Ab^	53.30 ± 1.91^Ac^	44.90 ± 1.76^Ab^	48.46 ± 1.72^Ae^	58.55 ± 1.58^Ad^
7	21.30 ± 1.2^Ba^	41.35 ± 1.06^Ab^	40.12 ± 2.18^Bb^	48.52 ± 2.55^Ac^	41.56 ± 1.68^Ab^	45.59 ± 2.17^Bd^	51.00 ± 2.41^Abc^
14	19.05 ± 0.62^Ba^	30.95 ± 0.75^Bb^	34.75 ± 2.82^Cbc^	38.00 ± 1.73^Bb^	31.00 ± 1.70^Bb^	34.55 ± 0.78^Cbc^	33.50 ± 1.37^Bbc^
21	14.12 ± 1.47^Ca^	27.92 ± 2.09^Cb^	31.55 ± 2.24 ^Ccb^	35.04 ± 1.53^Bb^	28.25 ± 1.56^Bc^	32.60 ± 1.74^Cb^	32.50 ± 1.05^Bb^

Note

Data are expressed as the means ± SD for three replicates. ^abcd^Different superscripts in the same row (*p* < .05). ^ABCD^ Different superscripts in the same column (*p* < .05).

## 4 DISCUSSION

In this study, OLP and OLE were added before and after fermentation at defined concentrations for the evaluation of organoleptic, microbial, and physicochemical properties of the final products.

There are some published reports about the addition of OLE to various foods and its effects on the sensory properties of the final products. Our results are in agreement with those reported by Peker and Arslan (2016), who observed that low‐fat apricot yoghurt enriched with various concentrations of OLE showed the acceptable sensory evaluation. A related study was designed to investigate the effect of OLE on the sensory evaluation of yoghurt during the 21‐day refrigerated storage. Results showed that increasing concentrations of OLE (0.2%, 0.4%) created a favorable taste in milk and yoghurt. However, when 0.6% of OLE was added, the sensory evaluation was not satisfactory (Marhamatizadeh et al., 2013). Tavakoli et al. reported that olive leaf phenolics encapsulated with nanoliposomes can be added to food products such as yoghurt to increase their nutritional value and public acceptance (without undesirable effects on their sensory characteristics) (Tavakoli et al., 2018). Difonzo et al. investigated the sensory aspects of nonthermally stabilized olive‐based pâté fortified with OLE at concentrations of 0.5 and 1 mg/kg. No sensory defects were perceived in all samples, even if a more intense typical olive flavor was perceived in samples containing OLE, compared to those containing butylated hydroxytoluene (BHT) and control samples (Difonzo et al., 2019). A related study was conducted to investigate the effect of incorporating OLE (1%) with or without tannic acid (TA; 0.02%), on the quality characteristics and shelf‐life extension of raw ground beef patties. Results showed that OLE and TA can be incorporated into beef patties without having a detrimental effect on the product quality. Moreover, during refrigerated storage, panel preference for treated samples was higher than that for untreated control, indicating that both OLE and TA were potent preservatives having a better function, essential for maintaining the sensory attributes of meat products (Moawad et al., 2017). Baker and Biol (2014) reported that addition of 1% OLE to lamb patties had significantly better overall acceptability compared to that of untreated or other treated groups. Based on the results of the current study and previous studies, it can be concluded that adding OLE (up to 3%) did not have any adverse effect on the sensory properties of the enriched food. Yoghurt fortified with different concentrations of tomato juice (0, 50, 100, 150, and 200 w/w%) was prepared, and results showed that the sample fortified with 50% tomato juice had the best acceptance in respect of the sensory evaluation of all the fortified yoghurt samples (Ademosun et al., 2019).

The results obtained from the current study are in agreement with those reported by Zoidou et al., in which the enrichment of yoghurt with OLE and pure oleuropein did not make a change in the populations of lactic acid bacteria when compared to those of the control (Zoidou et al., 2014). The counts of yoghurt bacteria slightly increased and then decreased during cold storage in a similar manner in control and enriched yoghurt samples, indicating that the enrichment did not affect their viability. In contrast with our findings, Georgakouli et al. reported that population of lactic acid bacteria (LAB) and production of lactate in yoghurt were significantly enhanced after the addition of olive polyphenols, contrary to the population of yeasts and molds (Georgakouli et al., 2016). In a related study, the effect of OLE (0.2%, 0.4%, and 0.6%) on yoghurt samples was investigated by Marhamatizadeh et al. (2013). They reported that the growth rate of bacteria was increased by increasing the concentration of OLE and reached the desired acidity at a shorter period. Moreover, the refrigerated samples containing 0.6% OLE powder possessed the highest count of bacteria, during 21 days of storage. In a study, yoghurt fortified with chickpea flour, which is a rich source of protein, promoted the growth of starter culture bacteria during yoghurt making and decreased of the incubation time (Chen et al., 2018). Yoghurt fortified with five different vegetable oils, including *Camelina sativa*, raspberry, black currant, and Echium plantagineum, did not influence the viability of lactic acid bacteria, which were higher than 10^7^ cfu/g at 21 days of storage (Dal Bello et al., 2015). Yoghurt containing olive leaf hot water extract (0, 0.1%, 0.2%, and 0.4% (w/v)) stored for 15 days at 4°C showed no significant effect on the population of lactic acid bacteria when compared to that of control yoghurt (Cho et al., 2020). Yoghurt supplemented with different concentrations (0%, 0.2%, 0.5%, and 1%) of lotus leaf (LL) powder showed LAB counts over 7.0 Log cfu/g for all yoghurt samples. The LAB counts increased with increasing LL concentrations, but not significantly (*p* > .05) (Kim et al., 2019). Also, yoghurt fortified with red ginseng extract (0.5%, 1%, 1.5%, and 2%) was produced and stored for 31 days at 4°C. The results showed that during fermentation, the viable cell counts of LAB did increased, but increase of red ginseng extract concentration resulted in a decrease of LAB cell counts (Jung et al., 2016). In similar studies, the addition of hazelnut skin (Bertolino et al., 2015) and freeze‐dried stevia to the yoghurt had suggested a significant effect on pH and TTA of the final product relative to control samples. However, adding oleuropein, which is a natural antioxidant (Zoidou et al., 2014), and tea (Jaziri et al., 2009) resulted in the same pattern between the pH of the enriched yoghurt and control samples. A similar result was reported by Cho et al. (2020); pH and TTA values of yoghurt containing olive leaf hot water extract gradually decreased and increased during storage, respectively. In addition, yoghurt fortified with lotus leaf powder showed increase of TTA and decrease of pH during 15 days of storage (Kim et al., 2019). Yoghurt supplemented with green olive powder (1%, 3%, and 5%) showed an increase of TTA and a decrease of pH during 15 days of storage (Cho et al., 2017). In a similar research conducted by Zoidou et al. (2014), oleuropein was detected in yoghurt enriched with pure oleuropein.

When OLP and OLE were added to the samples at the pre‐fermentation stage, further reduction of phenolic compounds was observed, which may be related to the activity of starter bacteria during fermentation. Similarly, the starter bacteria may use these components as carbon or energy source. Furthermore, the production of lactic acid during fermentation may lead to degradation of unstable phenolic compounds at acidic pH in the enriched yoghurt samples. Cho et al., (2020) prepared fortified yoghurt containing different olive leaf hot water concentrations, in which oleuropein was detected as the most abundant phenolic compound by HPLC.

Also, yoghurt fortified with phenolic compounds extracted from strawberry press residues was studied, and results showed that TPC obtained was 4640.0 ± 23.93 mg GAE/100 g dry extract (Ivanov & Dimitrova, 2019). The TPC increased when the yoghurt sample was enriched with different additives, including, freeze‐dried stevia (Carvalho et al., 2018) and peppermint, dill, basil (Amirdivani & Baba, 2011), when compared to the blank yoghurt. The TPC values of yoghurt fortified with red cactus pear peel powder and its mucilage powder were 348.0 ± 4.8 mg GAE/100 g and 410.6 ± 3.9 mg GAE/100 g, respectively (Hernández‐Carranza et al., 2019). Total phenol content (TPC) of yoghurt fortified at 0%, 1%, 3%, and 5% of green olive powder was obtained 4.30, 4.52, 5.85, and 6.96 (mg GAE/kg), which on day 15 of storage, TPC caluculated 3.67, 4, 4.82 and 5.60 (mg GAE/kg), respectively (Cho et al., 2017). In a similar report by Cho et al. (2020), the TPC of yoghurt fortified with different concentrations of olive leaf hot water extract increased with increasing concentrations of added olive leaf hot water extract and decreased with the increase of storage duration. The antioxidant activity percentage of yoghurt fortified with polyphenol‐enriched extracts from strawberry press residues was 2427 ± 5.00 µmol/TEg (Carvalho et al., 2018). Hernández‐Carranza et al. (2019) reported that yoghurt fortified with 5.5% of red cactus pear peel and 7.5% of its mucilage contained the highest concentrations of bioactive compounds and exhibited the highest antioxidant capacity (AC). AC percentage for red cactus pear peel fortified yoghurt was 1859.6 ± 64.7 mg ascorbic acid/100 g and for its mucilage, it was 3082.1 ± 99.3 mg ascorbic acid/100 g. The antioxidant activity percentage of yoghurt samples fortified with olive leaf hot water extract was reduced during storage and the increase of olive leaf hot water extract concentration led to an increased antioxidant activity (Cho et al., 2020). The DPPH radical scavenging activity of yoghurt fortified with green olive leaf powder decreased after a storage of 14 days, and also an increase in antioxidant activity percentage was observed with increasing green olive leaf powder concentrations (Cho et al., 2017).

## 5 CONCLUSIONS

Olive leaf powder and OLE are the rich sources of phenolic compounds; OLP is a healthy raw material that contains nutraceutical compounds. Yoghurt supplemented with OLP and OLE can be classified as a functional food. Adding OLP or its extract (OLE) during the formulation of yoghurt can affect the antioxidant activity, acidification rate, total phenol content, pH, and sensory properties. The viability of *L*. *delbrueckii* subsp. *bulgaricus* increased with the increase of OLP and OLE concentrations, but in the case of *S*. *thermophilus,* no changes in their cell counts were observed. The viability of starter bacteria did not decrease during the 21 days of storage. Both OLP and OLE prolonged the shelf life and antioxidant properties of the yoghurt; thus, OLP and OLE can be used as rich sources of phenolic compounds for the enrichment of milk and milk products, which are consumed daily by humans. In addition, yoghurt enriched with OLP or OLE can be considered as the commercial nutraceutical products.

## CONFLICT OF INTEREST

There is no conflict of interest regarding the publication of this manuscript.

## ETHICAL APPROVAL

This study does not involve any human or animal testing.

## Data Availability

The data that support the findings of this study are available from the corresponding author upon reasonable request.
